# Peptide TNIIIA2 Derived from Tenascin-C Contributes to Malignant Progression in Colitis-Associated Colorectal Cancer via β1-Integrin Activation in Fibroblasts

**DOI:** 10.3390/ijms20112752

**Published:** 2019-06-05

**Authors:** Motomichi Fujita, Yuka Ito-Fujita, Takuya Iyoda, Manabu Sasada, Yuko Okada, Kazuma Ishibashi, Takuro Osawa, Hiroaki Kodama, Fumio Fukai, Hideo Suzuki

**Affiliations:** 1 Department of Molecular Patho-Physiology, Faculty of Pharmaceutical Sciences, Tokyo University of Science, 2641 Yamazaki, Noda, Chiba 278-8510, Japan; motomichi.f@gmail.com (M.F.); yuka02079@gmail.com (Y.I.-F.); ssdy1321.tus@gmail.com (M.S.); bitawan850@gmail.com (Y.O.); k.ishibashi0604@gmail.com (K.I.); ohhhhhhhxx.w.am@gmail.com (T.O.); 2 Department of Pharmacy, Faculty of Pharmaceutical Sciences, Sanyo-Onoda City University, 1-1-1 Daigaku-Doori, Sanyo-Onoda, Yamaguchi 756-0884, Japan; iyoda@rs.socu.ac.jp; 3 Faculty of Science and Engineering, Saga University, 1 Honjo-machi, Saga-city, Saga 840-8502, Japan; hiroaki@cc.saga-u.ac.jp; 4 Translational Research Center, Research Institutes for Science and Technology, Tokyo University of Science, 2641 Yamazaki, Noda, Chiba 278-8510, Japan; hideoszk@md.tsukuba.ac.jp; 5 Department of Gastroenterology and Hepatology, Institute of Clinical Medicine, University of Tsukuba Graduate School, Tsukuba, Ibaraki 305-8575, Japan

**Keywords:** tenascin-C, colitis-associated colorectal cancer, β1-integrin, cancer-associated fibroblast, AOM/DSS model

## Abstract

Inflammatory bowel diseases increase the risk of colorectal cancer and colitis-associated colorectal cancer (CAC). Tenascin-C, a matricellular protein, is highly expressed in inflammatory bowel diseases, especially colorectal cancer. However, the role of tenascin-C in the development of CAC is not yet fully understood. We previously showed that a peptide derived from tenascin-C, peptide TNIIIA2, induces potent and sustained activation of β1-integrin. Moreover, we recently reported that peptide TNIIIA2 promotes invasion and metastasis in colon cancer cells. Here, we show the pathological relevance of TNIIIA2-related functional site for the development of CAC. First, expression of the TNIIIA2-containing TNC peptides/fragments was detected in dysplastic lesions of an azoxymethane/dextran sodium sulfate (AOM/DSS) mouse model. In vitro experiments demonstrated that conditioned medium from peptide TNIIIA2-stimulated human WI-38 fibroblasts induced malignant transformation in preneoplastic epithelial HaCaT cells. Indeed, these pro-proliferative effects stimulated by peptide TNIIIA2 were abrogated by peptide FNIII14, which has the ability to inactivate β1-integrin. Importantly, peptide FNIII14 was capable of suppressing polyp formation in the AOM/DSS model. Therefore, tenascin-C-derived peptide TNIIIA2 may contribute to the formation of CAC via activation of stromal fibroblasts based on β1-integrin activation. Peptide FNIII14 could represent a potential prophylactic treatment for CAC.

## 1. Introduction

The association of chronic inflammation with the pathogenesis of numerous malignancies has been well documented. In particular, it has been established that patients with inflammatory bowel diseases (IBDs), including ulcerative colitis and Crohn’s disease, have an increased risk of developing colorectal cancer, or colitis-associated colorectal cancer (CAC) [1,2,3]. Unlike sporadic colorectal cancer, the development of CAC does not display an adenoma-carcinoma sequence, but rather an inflammation-dysplasia-carcinoma sequence [4,5]. However, the molecular basis for the development of CAC remains unclear.

Tenascin-C (TNC), one of the typical matricellular proteins, is highly expressed in inflammatory lesions and tumor tissues [6,7]. A number of studies have also reported that upregulated expression of TNC occurs in ulcerative colitis, Crohn’s disease, and colon cancer [8,9,10,11]; however, the extent of the role of TNC in the pathogenesis of these diseases is not fully understood. We previously found that TNC harbors a cryptic functional site composed of the amino acid sequence YTITIRGV, which becomes active following proteolytic cleavage [12]. A 22-mer TNC peptide containing this functional sequence, termed peptide TNIIIA2, has a potent and sustained ability to activate β1-integrins [13]. Since this active sequence resides within the cancer-associated alternative splicing domain—fibronectin type III repeat A2 (FNIII-A2)–of the TNC molecule [14], it is presumed that the β1-integrin activation induced by TNIIIA2-containing TNC peptides/fragments may contribute to the pathogenesis of cancer. Recently, peptide TNIIIA2 was shown to act directly on colon cancer cells to promote their in vitro ability to invade through enhanced secretion of matrix metalloproteinase [15]. Furthermore, an in vivo experiment using a spontaneous metastasis model showed that peptide TNIIIA2 is also involved in the metastasis of colon cancer cells to the lung. On the other hand, we also found previously that a peptide derived from fibronectin, termed peptide FNIII14, induces inactivation of β1-integrins [16]. Interestingly, peptide FNIII14 has the ability to abrogate cell regulation by peptide TNIIIA2 [17].

In the present study, immunohistochemical analysis first showed high expression of TNIIIA2-containing TNC peptides/fragments in the stromal area of dysplastic lesions in AOM/DSS mice. Based on this observation, in vitro experiments focused on the effects of β1-integrin activation on both preneoplastic epithelial cells and stromal fibroblasts. Interestingly, the results suggested that the activation of β1-integrins by peptide TNIIIA2 does not have a significant direct effect on preneoplastic epithelial cells. However, stromal fibroblasts stimulated by peptide TNIIIA2 secreted a humoral factor(s) that induced malignant transformation in premalignant cells. Peptide FNIII14 suppressed both the in vitro effects of peptide TNIIIA2 and polyp formation in the AOM/DSS model, suggesting that the activation of β1-integrins is an important target for the prevention of IBDs including CAC. Peptide FNIII14, a unique factor that inactivates β1-integrins, may be a promising agent for the chemotherapeutic treatment of CAC.

## 2. Results

### 2.1. TNC/TNIIIA2 Expression in a Murine AOM/DSS Model

**Figure 1:**
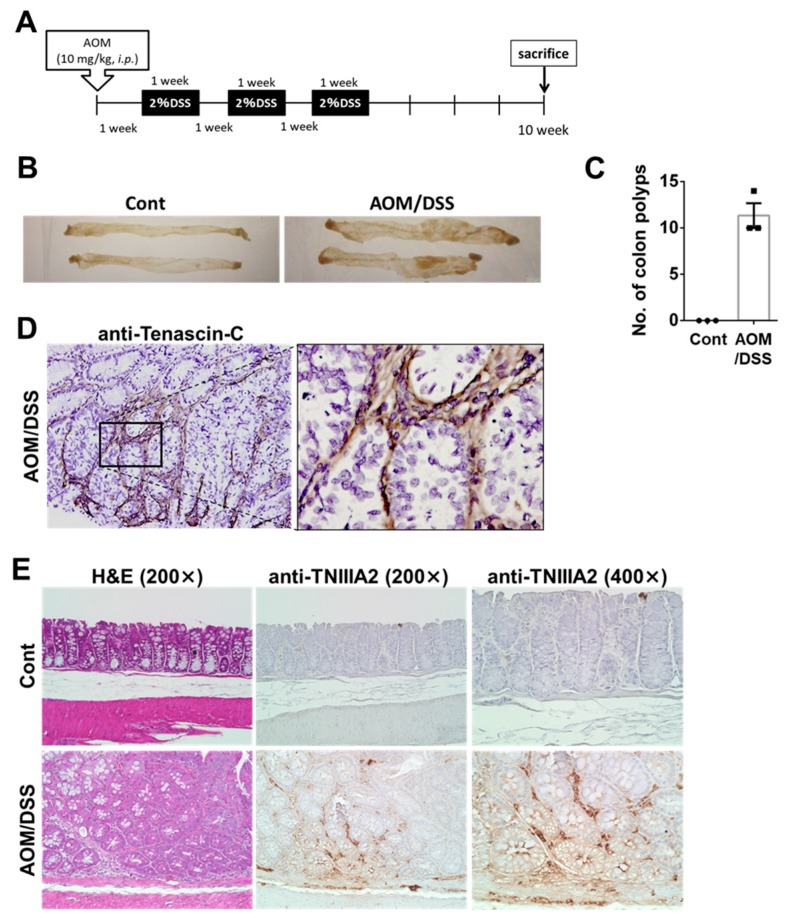
TNC/TNIIIA2 expressed in murine AOM/DSS colitis-associated carcinoma model. (**A**) Schematic of AOM and DSS administration to mice. (**B**) Macroscopic view of the resected colon in control and AOM/DSS-treated mice. (**C**) Tumor incidence in the AOM/DSS model. Dot plots show the number of colon polyps of individual mice in each group with bars representing means ± standard deviation. (**D**,**E**) Paraffin-embedded colon sections were stained with hematoxylin and eosin (H&E), TNC, and TNIIIA2 (original magnification 200× and 400×).

A well-characterized CAC model in ICR mice was generated by the administration of AOM and DSS, according to conventional methods [18] (Figure 1A). This model displayed dysplasia and adenomatous polyps 10 weeks after the first AOM injection (Figure 1B,C). We analyzed the expression levels of TNC and TNC fragments in the colon tissues of both untreated control mice and AOM/DSS-treated mice. In this immunohistochemical analysis, we used two kinds of antibodies: anti-TNC mAb recognizing the epidermal growth factor-like repeats present in all variants of TNC and anti-TNIIIA2 polyclonal Ab raised against the proadhesive amino acid sequence (YTITIRGV), which can detect TNIIIA2-containing TNC peptides/fragments. As expected, a high expression of TNC was detected in colonic tissues of AOM/DSS mice, using anti-TNC mAb (Figure 1D) [19]. TNIIIA2-containing TNC peptides/fragments recognized by anti-TNIIIA2 Ab were also detected in dysplastic lesions in the mucosa of AOM/DSS-treated mice (Figure 1E). It appeared that immunostaining for both TNIIIA2 and TNC show prominent localization around colon stromal cells. Thus, TNIIIA2-containing TNC peptides/fragments were shown to be expressed in dysplastic lesions of AOM/DSS mice. It is possible that β1-integrin activation induced by TNIIIA2-containing TNC peptides/fragments influences the development and progression of CAC.

### 2.2. Paracrine Factor(s) Released from Fibroblasts in Response to Stimulation of Peptide TNIIIA2 Induces Dysregulated Proliferation in Premalignant and Malignant Cells

**Figure 2:**
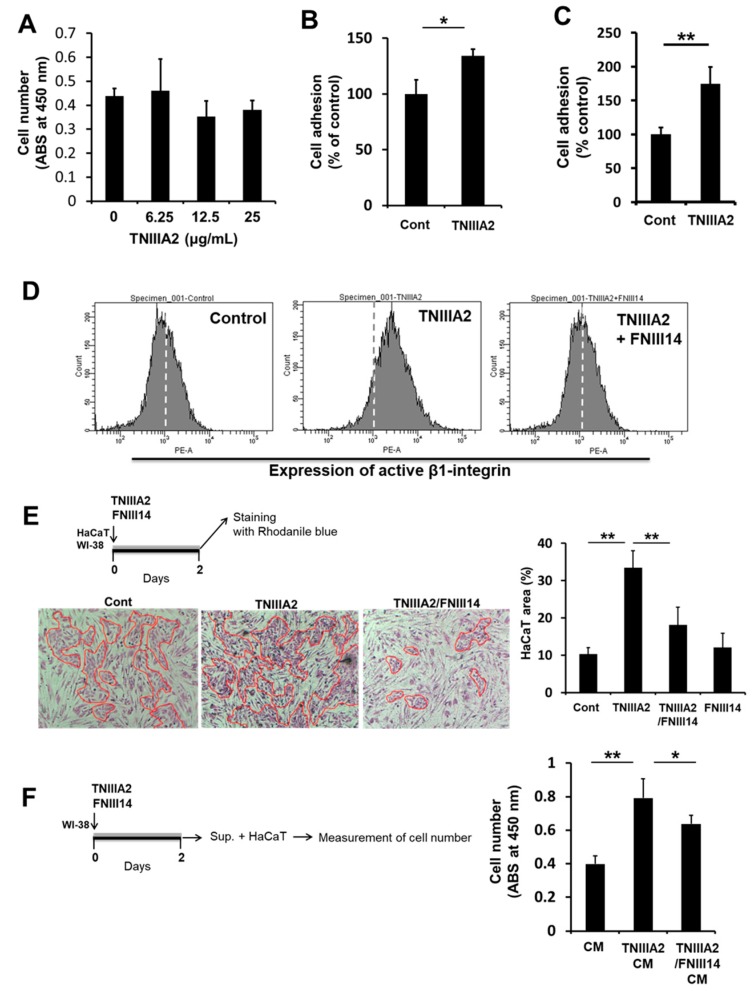
Peptide TNIIIA2 indirectly accelerated the proliferation of preneoplastic epithelial HaCaT cells via stimulation of WI-38 fibroblasts. (**A**) Effect of peptide TNIIIA2 (25 μg/mL) on the proliferation of HaCaT cells. Cells were treated with peptide TNIIIA2 (25 μg/mL) at the indicated concentrations. After 2 days, the number of cells was evaluated by WST-8 assay. (**B**) HaCaT cells were seeded with or without peptide TNIIIA2 (25 μg/mL) into 96-well plates coated with fibronectin (1.0 μg/mL). Adhered cells were measured by crystal violet staining and counted under a microscope. (**C**) WI-38 cells were seeded with or without peptide TNIIIA2 (25 μg/mL) into 96-well plates coated with fibronectin (1.0 μg/mL). Adhered cells were measured by crystal violet staining and counted under a microscope. (**D**) WI-38 cells were incubated with or without peptide TNIIIA2 (25 μg/mL) in the presence or absence of peptide FNIII14 (50 μg/mL) for 30 min. Activation status of β1-integrin was evaluated by flow cytometry using antibody AG89 recognizing the active β1-integrin conformation-specific epitope. (**E**) HaCaT cells were co-cultured with WI-38 in the presence or absence of peptide TNIIIA2 (25 μg/mL), peptide FNIII14 (50 μg/mL) or their combination for 2 days as described in Materials and Methods. Cells were stained with 1% Rhodanile blue for epithelial cells staining. Proliferation of HaCaT cells were evaluated as described in Materials and Methods. (**F**) WI-38 cells were treated in the absence or presence of peptide TNIIIA2 (25 μg/mL) and peptide FNIII14 (50 μg/mL) for two days, and the culture supernatants were collected as conditioned medium (“CM”: control, “TNIIIA2 CM”: cultured with TNIIIA2, “TNIIIA2/FNIII14 CM”: cultured with TNIIIA2 in the presence of FNIII14). Preneoplastic HaCaT cells were then cultured with each of these conditioned media. Two days later, the number of viable cells was evaluated by WST-8 assays, as described in the ‘Materials and Methods’. Data represent the means ± standard deviation of triplicate determinations, * *p* < 0.05, ** *p* < 0.01.

Based on the immunohistochemistry results showing that TNIIIA2-containing TNC peptides/fragments were localized within dysplastic lesions of AOM/DSS-treated mice, the effects of β1-integrin activation on human preneoplastic epithelial cell line HaCaT was investigated using peptide TNIIIA2 as a representative of TNIIIA2-containing TNC peptides/fragments. However, peptide TNIIIA2 showed no remarkable effects on the proliferation of HaCaT cells (Figure 2A), although adhesion to the fibronectin substrate was significantly promoted (Figure 2B). Considering the involvement of stromal fibroblasts in cancer pathology [20,21], we next investigated whether fibroblastic cells treated with TNIIIA2 can affect the malignant transformation of preneoplastic HaCaT cells. Human normal fibroblasts, WI-38 cells, were used in the following experiments. First, it was verified that peptide TNIIIA2 promoted the adhesion of WI-38 cells to a fibronectin substrate (Figure 2C), in response to activation of β1-integrins by TNIIIA2 (Figure 2D), as determined by flow cytometry using a monoclonal antibody recognizing an active conformation-specific epitope of the β1-integrins. Therefore, HaCaT cells were then co-cultured with WI-38 cells in the presence or absence of peptide TNIIIA2, and the proliferation of HaCaT cells was evaluated by selective staining of epithelial cells with Rhodanile blue. As shown in Figure 2E, co-culture with WI-38 fibroblasts markedly increased the area occupied by HaCaT cells in the presence of peptide TNIIIA2 (represented as “TNIIIA2” in Figure 2E), compared to when it was absent (represented as “Cont”). To clarify the involvement of β1-integrin activation in the promotion of cell growth by peptide TNIIIA2 in this co-culture system, the effect of peptide FNIII14, which induces inactivation in β1-integrins (Figure 2D), was examined. As expected, the promotion of HaCaT cell proliferation by peptide TNIIIA2 in the co-culture system was prevented by the addition of FNIII14 (represented as “TNIIIA2/FNIII14” in Figure 2E). Peptide FNIII14 alone showed no noticeable effect on HaCaT cell proliferation in the co-culture system (represented as “FNIII14” in Figure 2E). To investigate the role of WI-38 cells in promoting HaCaT cell proliferation in the co-culture system, the effects of WI-38 fibroblasts in conditioned medium (CM) on HaCaT cell proliferation were analyzed. WI-38 fibroblasts were cultured with or without peptide TNIIIA2. After 48 h, HaCaT cells were cultured in CM (Figure 2F). CM from WI-38 cells stimulated with peptide TNIIIA2 (represented as “TNIIIA2 CM”) remarkably enhanced the proliferation of HaCaT cells, compared to CM from WI-38 cells cultured without peptide TNIIIA2 (represented as “CM”) (Figure 2F). This peptide TNIIIA2-mediated cell proliferation via WI-38 fibroblasts was also partially suppressed by peptide FNIII14 (Figure 2F).

**Figure 3:**
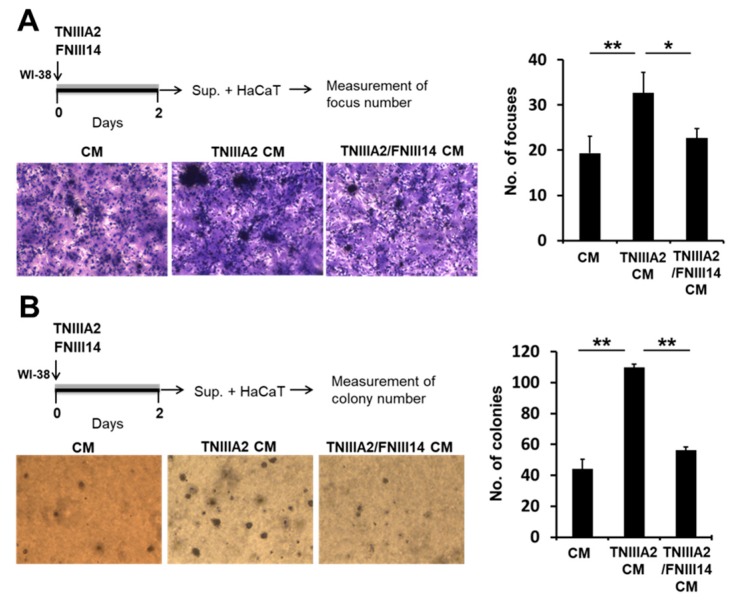
Peptide TNIIIA2-induced malignant progression in preneoplastic epithelial HaCaT cells. Conditioned medium from peptide TNIIIA2-stimulated WI-38 fibroblasts enhanced the proliferation/survival in preneoplastic epithelial HaCaT cells. In panels (**A**) and (**B**), WI-38 cells were treated for 2 days in the absence or presence of peptide TNIIIA2 (25 μg/mL) and peptide FNIII14 (50 μg/mL), respectively, and the culture supernatants were collected as conditioned medium, as described in the legend of Figure 2F. Preneoplastic HaCaT cells were subjected to the focus formation assay (**A**) or colony formation assay (**B**) using these conditioned media, as described in the “Materials and Methods”. Data represent the means ± standard deviation of triplicate determinations, * *p* < 0.05, ** *p* < 0.01.

Noticeably, CM from peptide TNIIIA2-stimulated WI-38 cells induced the formation of cell foci consisting of multilayered HaCaT cells (Figure 3A). Furthermore, CM from peptide TNIIIA2-stimulated WI-38 cells also promoted anchorage-independent proliferation in HaCaT cells, as shown by a soft agarose colony formation assay (Figure 3B). This phenotypic change in HaCaT cells was also suppressed by peptide FNIII14 (Figure 3A,B). These results indicated that peptide TNIIIA2 acts on WI-38 to stimulate the release of a paracrine factor(s), which induces malignant transformation of preneoplastic epithelial HaCaT cells. Since the effect of peptide TNIIIA2 was reversed by peptide FNIII14 with an opposing effect on integrin activation, the enhanced proliferation of HaCaT cells in both anchorage-dependent and -independent manners by peptide TNIIIA2 seems to be due to the activation of β1-integrin.

**Figure 4:**
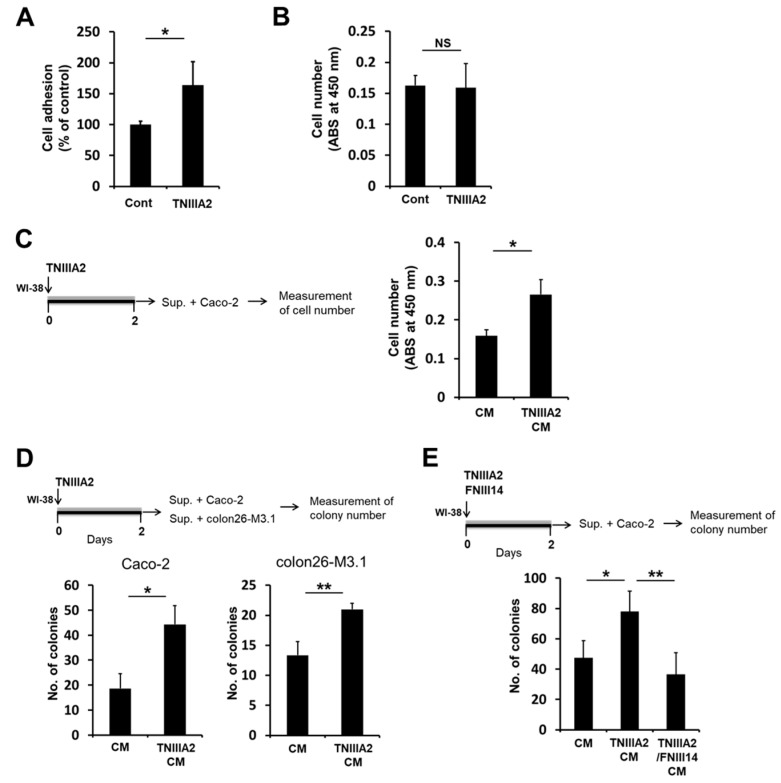
Conditioned medium from peptide TNIIIA2-stimulated WI-38 fibroblasts enhanced proliferation/survival in intestinal epithelial carcinoma cells. (**A**) Caco-2 cells were seeded with or without peptide TNIIIA2 (25 μg/mL) into 96-well plates coated with fibronectin (1.0 μg/mL). Adhered cells were measured by crystal violet staining and counted under a microscope. (**B**) The effect of peptide TNIIIA2 (25 μg/mL) on the proliferation of Caco-2 cells. Caco-2 cells were treated with peptide TNIIIA2 (25 μg/mL) for two days, and the number of cells was evaluated by WST-8 assay. In panels (**C**), (**D**), and (**E**), WI-38 cells were cultured for 2 days in the absence or presence of peptide TNIIIA2 (25 μg/mL) and peptide FNIII14 (50 μg/mL), and the culture supernatants (conditioned medium) were collected as described in the legend of Figure 2F. (**C**) Caco-2 cells were cultured for 2 days with “CM” or “TNIIIA2 CM”, and the number of viable cells was evaluated by WST-8 assay. (**D**) Caco-2 cells or colon26-M3.1 cells were subjected to the colony formation assay using “CM” or “TNIIIA2 CM”, as described in the ‘Materials and Methods’. (**E**) The effect of peptide FNIII14 on enhanced ability of colony formation by “TNIIIA2 CM”. Caco-2 cells were subjected to the colony formation assay using “CM”, “TNIIIA2 CM” or “TNIIIA2/FNIII14 CM). Data represent the means ± standard deviation of triplicate determinations, * *p* < 0.05, ** *p* < 0.01, NS not significant.

We next examined the effects of peptide TNIIIA2 on the neoplastic cell lines Caco-2 and colon26-M3.1. Similar to the effects on preneoplastic epithelial HaCaT cells, peptide TNIIIA2 promoted cell adhesion to the fibronectin substrate (Figure 4A) but did not directly affect the proliferation of human colorectal adenocarcinoma cell line Caco-2 (Figure 4B). CM from peptide TNIIIA2-stimulated WI-38 fibroblasts increased the proliferation of Caco-2 cells on the fibronectin substrate (represented by “TNIIIA2 CM” in Figure 4C). Furthermore, CM from peptide TNIIIA2-stimulated WI-38 fibroblasts strongly stimulated Caco-2 cell proliferation, also in an anchorage-independent manner (Figure 4D, left panel). Similar phenomena were observed for another colon cancer cell line, colon26-M3.1 (Figure 4D, right panel). Importantly, peptide FNIII14 suppressed the effects of peptide TNIIIA2-stimulated WI-38 fibroblasts (Figure 4E). These results suggest that a paracrine factor(s) from peptide TNIIIA2-stimulated WI-38 fibroblasts induces malignant progression in premalignant epithelial HaCaT cells as well as in overtly malignant cells.

### 2.3. Peptide FNIII14 Suppresses Polyp Formation in the AOM/DSS Model

**Figure 5:**
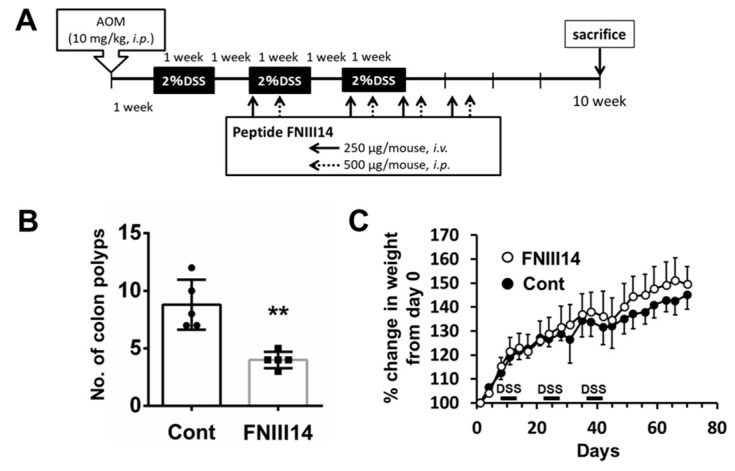
Peptide FNIII14 is capable of suppressing the incidence of colon polyps in the murine AOM/DSS colitis-associated carcinoma model. (**A**) Schematic of the administration of AOM, DSS, control peptide, and peptide FNIII14 to the mice. (**B**) The effect of peptide FNIII14 on the development of colitis-associated colorectal cancer in the AOM/DSS mouse model. Dot plots show the numbers of colon polyps of individual mice in each group with bars representing the means ± standard deviation, ** *p* < 0.01. (**C**) Body weight profile of the animals. Data represent the means ± standard deviation.

Using the AOM/DSS model, we investigated whether peptide FNIII14 exhibits a prophylactic or therapeutic effect on CAC (Figure 5A). The administration of peptide FNIII14 in mice significantly reduced the total number of polyps formed in the inner epithelial walls of the large intestinal tracts of the AOM/DSS mouse model (Figure 5B). There were no significant changes in body weight between the groups (Figure 5C). Taken together, β1-integrin activation by TNIIIA2-containing TNC peptides/fragments generated in inflammatory dysplasia lesions may be involved in the malignant transformation of preneoplastic cells and the malignant progression of colon cancer cells via activation of fibroblasts in the tumor microenvironment, and peptide FNIII14, which has the ability to neutralize TNIIIA2 activity, might lead to the suppression of CAC development and progression.

## 3. Discussion

TNC is highly expressed during inflammation and several malignant cancers and its expression levels are associated with a poor prognosis in cancer patients [6]. Furthermore, a high expression of TNC is considered to be a poor prognostic factor in colorectal cancer tissues [22]. Elevated expression of TNC has been reported in areas of ulceration in ulcerative colitis as well as in areas of stricture in Crohn’s disease [23]. Thus, it seems that TNC contributes to the development and/or progression of CAC. Identification of the role and the functional sites of TNC molecule responsible for CAC would enable the design of inhibitors with therapeutic potential for these diseases. More recently, TNC derived from intestinal myofibroblasts was shown to promote the onset of CAC in an AOM/DSS model [19]. In addition, extracellular matrix (ECM) remodeling is often enhanced in these pathological regions and proteolytic cleavage of ECM proteins including TNC is carried out by several inflammatory proteinases [24,25,26]. Indeed, elevated levels of several matrix metalloproteinases (MMPs) have been observed in IBD and are associated with IBD disease activity [27], indicating that the degradation and remodeling of the ECM, including TNC, may occur at a high level in IBD and during the development of CAC. This study supports earlier findings by demonstrating that high expression levels of TNC and the TNIIIA2-containing TNC peptides/fragments are present in dysplastic lesions in the AOM/DSS mouse model. Furthermore, our results suggest that TNIIIA2-containing TNC peptides/fragments are involved in the pathogenesis of CAC via potentiated and sustained activation of β1-integrin. Thus, β1-integrin activation by TNIIIA2-containing TNC peptides/fragments is an important target process for the prevention of CAC development at the cellular level. Furthermore, peptide FNIII14 suppressed not only dysregulated survival/proliferation of preneoplastic epithelial HaCaT cells but also the incidence of polyps in an AOM/DSS mouse model. Peptide FNIII14, which has the ability to neutralize the detrimental effects of TNIIIA2-containing TNC peptides/fragments by β1-integrin activation, would be a promising agent for the prevention of CAC development and progression.

A number of studies have indicated that cells in the tumor microenvironment, such as tumor stroma fibroblasts, and senescent and immune cells, are able to influence tumor progression. Among them, fibroblasts, specifically cancer-associated fibroblasts, are key determinants of carcinogenesis and cancer progression [20,21]. Sasaki and colleagues reported that the incidence of colon tumors is suppressed in CCL3- or CCR5-deficient mice treated with AOM/DSS and coincides with lower accumulation of fibroblasts in dysplastic lesions compared with wild-type mice [28]. They also showed that fibroblasts express HB-EGF to promote the proliferation of tumor cells in colitis-associated colorectal carcinogenesis in mice [28]. Moreover, fibroblast-derived epiregulin increases the proliferation of intestinal epithelial cells via the activation of ERK signaling pathway, which enhances the growth of CAC [29]. Thus, cancer-associated fibroblasts might be responsible for the development and progression of CAC. In addition, there is increasing evidence that a high expression of TNC along with other CAF markers in tumor stroma correlates with poor prognosis in several malignancies, such as esophageal squamous cell carcinoma [30], breast ductal carcinoma [31], prostate cancer [32], and colorectal cancer [33], indicating that TNC activates CAF to promote tumor progression. Here, we have shown that conditioned medium from peptide TNIIIA2-activated fibroblasts enhanced the survival/proliferation of both preneoplastic cells and colon cancer cells. Further investigations are required to identify the paracrine factor(s) that induces focus formation and anchorage-independent proliferation in preneoplastic epithelial HaCaT cells. In addition, an understanding of the signaling pathway by which β1-integrin activation triggers the secretion of this paracrine factor would contribute to the development of prophylactic agents for CAC. Further studies are also required to determine whether peptide FNIII14 actually attenuates the activation of fibroblasts in tumor microenvironment via inactivation of β1-integrins.

Accumulating evidence indicates that increased ECM stiffness is associated with the malignancy of tumors. A recent study demonstrated that glioblastoma aggression correlates with the stiffness of a TNC-enriched ECM [34]. More recently, Barnes et al. reported that glioblastoma cells expressing an auto-clustering V737N mutated β1-integrin exhibit potentiated mechanosignaling, increased ECM stiffness, while promoting tumor malignancy [35]. Increased ECM stiffness has also been observed in the stricture area of Crohn’s disease [36]. Increased ECM stiffness augments adhesive properties, such as the formation of focal adhesion and actin stress fibers of colonic fibroblasts [36]. We previously reported that peptide TNIIIA2 has the ability to induce potent and sustained activation as well as clustering of β1-integrin [12,13]. Thus, the potent activation of β1-integrin by the TNIIIA2-containing TNC peptides/fragments may contribute to CAC development by increasing the stiffness of the ECM. Further investigations are needed to verify whether β1-integrin activation by peptide TNIIIA2 actually increases ECM stiffness.

## 4. Materials and Methods

### 4.1. Reagents

Human plasma fibronectin was purified as described previously [37]. Peptide TNIIIA2 and FNIII14 has been described previously [12,38]. Dextran sodium sulfate (DSS) and Rhodanile blue were purchased from Sigma-Aldrich (Tokyo, Japan). Azoxymethane (AOM) was purchased from Wako Pure Chemicals (Tokyo, Japan).

### 4.2. Murine AOM/DSS Colitis-Associated Carcinoma Model

All the animal procedures were approved by the Institutional Animal Care and Use Committee of Tokyo University of Science. To develop colitis-associated carcinoma, we challenged 6–8-week-old male ICR mice (Sankyo Laboratory Service, Tokyo, Japan) with the organotropic carcinogen AOM (10 mg/kg body weight, intraperitoneally). Following AOM, mice were treated with 7 days of 2% DSS in drinking water, followed by recovery for 7 days with regular drinking water. The seven days of 2% DSS followed by seven days of normal drinking water was repeated three times. Mice were then analyzed 71 days after the AOM injection, and colons were removed and flushed with PBS and cut longitudinally. The number of colon polyps was counted using a dissecting microscope. For administration of peptide FNIII14, mice were then intravenously injected with FNIII14 (250 μg/mouse) on days 22, 36, 43, and 49, and intraperitoneally injected with FNIII14 (500 μg/mouse) on days 24, 38, 45, and 51.

### 4.3. Cell Culture

Human diploid lung embryonic fibroblast cell line WI-38, which was obtained from RIKEN Bio Resource Center (Ibaraki, Japan), was maintained in EMEM medium (Nissui Pharmaceutical, Tokyo, Japan) supplemented with 10% FBS (SAFC Biosciences, MO, USA). Human preneoplastic epidermal keratinocytes HaCaT, which was kindly provided by Dr. Masuho Yasuhiko (Tokyo University of Science), was maintained in DMEM medium (Nissui Pharmaceutical) supplemented with 10% FBS. Human colorectal adenocarcinoma cell line Caco-2, which was kindly provided by Dr. Michihiro Mutoh (National Cancer Center Research Institute), was maintained in DMEM medium supplemented with 10% FBS. Mouse colon cancer cell line colon26-M3.1, which was kindly provided by Dr. Ikuo Saiki (University of Toyama), was maintained in RPMI1640 medium supplemented with 10% FBS. Cells were incubated in a 5% CO_2_ incubator at 37 °C.

### 4.4. Immunohistochemistry

The resected specimens were fixed in 10% formalin and embedded in paraffin. For antigen retrieval, the slides were incubated at 37 °C for 30 min in 0.2% trypsin. The paraffin-embedded tissue samples were cut to a 4 µm thickness and stained with hematoxylin and eosin. The following immunohistochemical reagents were performed with anti-TNC 4F10TT Antibody (IBL, Gunma, Japan) and anti-TNIIIA2 polyclonal antibody (IBL) and Histofine Sab-Po (Multi) kit (Nichirei Biosciences, Inc, Tokyo, Japan) according to the manufacturer’s instructions. Images were captured using a light microscope Olympus BX-53 (Olympus, Tokyo, Japan).

### 4.5. Cell Adhesion Assay

Adhesion to fibronectin was performed as described previously [13].

### 4.6. Flow Cytometry

The activation status of β1-integrins on the cells was evaluated by flow cytometric analysis using a monoclonal antibody that recognize the active conformation of β1-integrin (clone AG89; MBL, Aichi, Japan), as described previously [12].

### 4.7. 2D Co-Culture Experiments

WI-38 cells (2.5 × 10^4^ cells/well) were seeded on 24-well culture plates. After 24 h, HaCaT cells (4.0 × 10^4^ cells/well) suspended in a 1:1 mixture of DMEM-EMEM medium in the presence or absence of TNIIIA2 and/or peptide FNIII14 were overlaid. After 48 h, cells were fixed with 4% paraformaldehyde and stained with 1% Rhodanile blue for epithelial cells staining [39]. Images were captured and area of epithelial cells was measured by Motic Image Plus 2.2S (Shimadzu Rika, Kyoto, Japan).

### 4.8. Preparation of Conditioned Medium from WI-38 Cells

WI-38 cells (2.0 × 10^5^ cells/well) were seeded on 12-well culture plates coated with fibronectin (1.0 μg/mL) and then cultured with the EMEM medium without FBS in the presence or absence of peptide TNIIIA2 and/or peptide FNIII14. Two days later, culture supernatants were collected as conditioned media (CM) and stored at −80 °C until use.

### 4.9. Cell Proliferation Assay

Cells (8.0 × 10^3^ cells/well) were seeded on 96-well culture plates coated with fibronectin (1.0 μg/mL) and then cultured with medium in the presence or absence of peptide TNIIIA2, or CM prepared under various conditions. The number of viable cells was evaluated based on the WST-8 assay, as described previously [13].

### 4.10. Colony Formation Assay

Solution of a 1:1 mixture of 1.4% agar (BD Bioscience, NJ, USA) and 2× DMEM or RPMI 1640 growth medium was poured into 12-well plates. After solidification, HaCaT (1.0 × 10^4^ cells/well), Caco-2 (7.0 × 10^3^ cells/well), or colon26-M3.1 (4.0 × 10^3^ cells/well) suspended in CM prepared under various conditions containing 0.7% agar were overlaid on top of a base layer. After solidification of the top agar layer, CM prepared under the corresponding conditions was added. Media were changed every 4–5 days. After 10-14 days, colonies were stained with crystal violet and the number of them was counted for five randomly selected fields under the microscope at 20× magnification.

### 4.11. Focus Formation Assay

Cells (2.0 × 10^5^ cells/well) were seeded on 24-well culture plates coated with fibronectin (1.0 μg/mL) and cultured with CM prepared under various conditions. After 8-10 days, cells were fixed with 4% paraformaldehyde and stained with crystal violet. Images were captured and analyzed by Motic Image Plus 2.2S.

### 4.12. Statistical Analysis

Data are expressed as the mean ± standard deviation. Two-tailed Student *t* test or one-way ANOVA analysis was used to determine statistical differences. Values of *p* < 0.05 were considered significant.

## Abbreviations

IBD	inflammatory bowel disease
UC	ulcerative colitis
CD	Crohn’s disease
CAC	colitis-associated colorectal cancer
AOM	azoxymethane
DSS	dextran sodium sulfate
*i.p.*	intraperitoneal injection
EGF	epidermal growth factor
CM	conditioned medium
MMP	matrix metalloproteinase
ECM	extracellular matrix
HB-EGF	heparin-binding EGF-like growth factor
ABS	absorbance
Sup.	supernatant
